# In-Situ Doping B_4_C Nanoparticles in Mesophase Pitch for Preparing Carbon Fibers with High Thermal Conductivity by Boron Catalytic Graphitization

**DOI:** 10.3390/molecules27165132

**Published:** 2022-08-12

**Authors:** Yue Liu, Jiahao Liu, Jianxiao Yang, Xiao Wu, Jun Li, Kui Shi, Bo Liu, Ruixuan Tan

**Affiliations:** 1 Hunan Province Key Laboratory for Advanced Carbon Materials and Applied Technology, College of Materials Science and Engineering, Hunan University, Changsha 410082, China; 2 School of Chemistry and Biological Engineering, Changsha University of Science and Technology, Changsha 410114, China; 3 Key Laboratory of Bionic Engineering, Ministry of Education, Jilin University, Changchun 130022, China

**Keywords:** mesophase pitch, carbon fiber, graphitization

## Abstract

The boron carbide (B_4_C) nanoparticles doping mesophase pitch (MP) was synthesized by the in-situ doping method with tetrahydrofuran solvent, and the corresponding MP−based carbon fibers (CFs) were successfully prepared through the melt−spinning, stabilization, carbonization and graphitization processes. The structural evolution and properties of boron−containing pitches and fibers in different processes were investigated for exploring the effect of B_4_C on mechanical, electrical and thermal properties of CFs. The results showed that the B_4_C was evenly dispersed in pitch fibers to provide active sites of oxygen, resulting in a homogeneous stabilization and ameliorating the split−ting microstructures of CFs. Moreover, the thermal conductivity of B1−MP−CF prepared with 1 wt.% B_4_C increased to 1051 W/m•K, which was much higher than that of B0−MP−CF prepared without B_4_C (659 W/m•K). While the tensile strength of B_4_C−doped CFs was lower than that of pristine CFs. In addition, a linear relationship equation between the graphite microcrystallite parameter (I_D_/I_G_) calculated from Raman spectra and the thermal conductivity (λ) calculated according to the electrical resistivity was found, which was beneficial to understand the thermal properties of CFs. Therefore, the doping B_4_C nanoparticles in MP did play a significant role in reducing the graphitization temperatures due to the boron catalytic graphitization but decreasing the mechanical properties due to the introduction of impurities.

## 1. Introduction

Thermal conductivity is one of the most important properties of carbon fibers (CFs), and CFs with high thermal conductivity are widely used in industrial, aerospace and military applications [1,2]. As an essential method to improve thermal conductivity of CFs, graphitization is carried out under the protection of inert gas, where the non−carbon elements in the CFs are removed to achieve carbon enrichment (>99%). Considering, phonon vibration is the main way of heat transfer in CFs, the graphite microcrystallite size and orientation of CFs affect their thermal conductivity to a great extent. As mesophase pitch (MP) is prone to form the graphite microcrystallite with high orientation. It can explain why MP−based CFs (MPCFs) can achieve high thermal conductivity. However, MPCFs also require much high−temperature graphitization treatments over 2800 °C, which inevitably leads to high energy consumption and short equipment life. Therefore, it is necessary to enhance the thermal conductivity of MPCFs while reducing the graphitization temperature and improving their microstructure. In recent years, the catalytic graphitization method has been proven to be one of the most effective measures to increase the thermal conductivity and decrease the graphitization temperature of CFs [3]. Generally, the catalysts are mainly composed of non−carbon elements, especially non−metal elements and metal elements. Quite a few literatures have demonstrated that the most effective catalysts are boron and borides, whose boron atoms can replace carbon atoms of graphite microcrystallite during graphitization, eliminating the negative influence of residual catalyst on other properties [4]. At the same time, scholars have carried out detailed research on the boron catalytic graphitization mechanism. Lowell [5] found that boron would form boron carbide in carbon materials, and the formed solid solution of boron in graphite would lead to a series of reactions at high temperature, which were beneficial to the diffusion of boron atoms in carbon materials [6]. Then the diffusion boron atoms would replace the carbon atoms at the vertices of the graphite hexagonal network structure, preventing the boron atoms from being arranged disorderly in the interlayer structure. Consequently, the energy required for the catalytic reaction was reduced and the graphitization process was greatly accelerated [7,8]. These findings convincingly suggested that the boron and borides could exhibit an unprecedented catalytic graphitization effectiveness. However, the method on how to carry out the homogeneous distributions of boron and borides in fibers remains unclear. In fact, the type of added boron and the addition steps will affect the properties of final carbon materials.

Currently, there are several main ways to dope boron and borides into fibers. Firstly, the impregnation of CFs with boric acid and boron nitride suspensions is recognized as a common liquid phase doping method. Wang et al. [9] immersed CFs in boron nitride suspension to obtain the boron doped CFs. The results found that boron nitride could catalyze the graphitization of CFs. However, the boron content in fiber skin was much higher than that in the core. In the meantime, violent reaction would occur at high temperatures, resulting in the formation of defects and strength decline. The indirect introduction is another method. Wang et al. [10] completed the diffusion doping of boron by preparing B_4_C/graphite crucible and exposing the CFs to the boron vapor obtained from the high−temperature decomposition of B_4_C. The results showed that boron diffused into CFs at high temperatures and played a role in graphitization, thereby boron doped CFs with flake ordered graphite structure could be then obtained. However, the strong erosion of boron vapor not only changed the original structure of CF, but also produced more defects. Moreover, the vapor deposition is a relatively common method, which uses inert gas as a carrier to introduce boride into the graphitization furnace, and the boride was decomposed and deposited on the surface of CFs at high temperature. Although the catalyst could be evenly distributed and stable, its high cost is still an unavoidable drawback. In summary, the above methods mainly focus on doping the boron into the CFs to describe the catalytic graphitization of boron in the graphitization stage. Even if some methods can significantly improve the properties of CFs, the producing cost of CFs will be greatly increased.

Compared with the aforementioned methods, the in-situ doping method has obviously more advantages and higher feasibility. For example, Chen et al. [11] prepared the high modulus PAN−based CFs using the B_4_C−doped PAN as a precursor which was synthesized by in-situ polymerization of acrylonitrile monomer with B_4_C nanoparticles. It proved that the in-situ doped B_4_C indeed played a significant role in catalytic graphitization and improving microstructure of PAN−based CFs. Yu et al. [12] systematically studied the effect of B_4_C on the properties of graphite foam from MP. They found that adding B_4_C increased the graphitization degree and the thermal conductivity of graphite foam by 97% and 52.5%, respectively. Accordingly, boron and borides can excellently accomplish the objective of catalyzing graphitization and improve the fiber microstructure. This means that MPCFs with high thermal conductivity can be prepared at a low graphitization temperature by boron catalytic graphitization. In this work, the uniform distribution of boron carbide (B_4_C) nanoparticles in pitch fibers were achieved by the in-situ doping boron nanoparticles in MP and following melt−spinning process. The structural evolution and properties of boron−dopped fibers in the stabilization, carbonization and graphitization processes were investigated for exploring the effect of B_4_C nanoparticles on mechanical, electrical and thermal properties of MPCFs.

## 2. Materials and Methods

### 2.1. Materials

MP was a wide−area streamline feature with mesophase content over 90% and softening point of 285 °C, which was provided by Hunan Dongying Carbon Material Technology Co., Ltd., Changsha, China. B_4_C nanoparticles with 50 nm (CAS 12069−32−8, 99%) was purchased from Sigma−Aldrich (Shanghai, China) Trading Co., Ltd., Shanghai, China.

### 2.2. Preparation of B_4_C−Doped Mesophase Pitch−Based Carbon Fibers

The preparation progress of B_4_C−doped MPCFs were described as follows: Firstly, MP was grounded and sieved with a 60−mesh sieve. Then 25 g MP was dissolved in 250 g tetrahydrofuran (THF) solvent and added the B_4_C nanoparticles with different contents (0, 1, 5 wt.%), and the mixture was magnetic stirred at room temperature for 5 h. The B_4_C−doped MP was successfully prepared after the following evaporation and vacuum drying at 60 °C for 12 h. Secondly, 10 g B_4_C−doped MP was spun into pitch fibers (PFs) with a single−hole spinning apparatus (the length/diameter of spinneret is 0.4 mm/0.2 mm) at the spinning temperature of 350 °C and nitrogen pressure of 1.0 MPa. Thirdly, 3 g B_4_C−doped MP−derived PFs which were pre−cut into short fibers with length of 10 cm were stabilized at 270 °C for 1 h with a heating rate of 1 °C/min in a 200 mL/min air atmosphere to obtain the stabilized fibers (SFs) [13]. Finally, 2 g B_4_C−doped MP−derived SFs were subsequently carbonized at 1000 °C for 1 h with a heating rate of 5 °C/min in a 200 mL/min nitrogen atmosphere and furtherly graphitized at 2300, 2600, 2800 or 3000 °C for 10 min in a graphitization furnace to obtain the B_4_C−doped MP−derived CFs. The samples obtained from the different preparation process parameters were named as B*x*−MP, B*x*−MP−PF, B*x*−MP−SF, B*x*−MP−CF−*t*, respectively (*x* represented the adding B_4_C contents, *t* represented the carbonization and graphitization temperature).

### 2.3. Softening Point (SP) and Polarizing Optical Microscopy (POM)

The SP and POM photos of pitches were determined and observed by a CFT−100EX capillary rheometer (Shimadzu, Kyoto, Japan) and a BX53 polarizing microscope (Olympus, Tokyo, Japan).

### 2.4. Scanning Electron Microscope (SEM)

The morphology and microstructure of CFs were analyzed by a SU8010 SEM microscopy (Hitachi, Tokyo, Japan) with 5 kV.

### 2.5. Infrared Spectroscopy (FTIR)

The FTIR spectra of pitches and fibers were obtained using KBr disc technique in a Nicolet iS10 FTIR spectrometer (Thermo Fisher Scientific, Waltham, MA, USA). The ortho−substitution index (IOS) represents the fraction of aromatic rings with ortho substitutions and gives the relative size of aromatic molecules, which was defined as the following Equation (1). The C−H substitution index (I_CHS_) represents the fraction of aromatic carbons that are substituted with aliphatic (−CH_3_) or methylene (−CH_2_−) groups, which was defined as the following Equation (2) [14].

(1) $$I_{\mathrm{OS}}=\frac{Abs_{750}}{Abs_{750}+Abs_{814}+Abs_{840}+Abs_{880}}$$

(2) $$I_{\mathrm{CHS}}=\frac{Abs_{2920}}{Abs_{3050}+Abs_{2920}}$$

### 2.6. Raman

The Raman spectra of pitches and fibers were recorded by a DXR2 Raman Microscope (Thermo Fisher Scientific) and the information of G, D, D’ and A peaks were obtained by the Lorentz fitting to calculate the area ratio of I_D_/I_G_, I_D’_/I_G_ and I_A_/I_G_, which could be used to quantitatively evaluate the graphitized degree, structural order and the content of functional groups of CFs. Moreover, their graphite microcrystalline parameters of plane size (*La*, nm) was calculated according to the following Equation (3) [15].

(3) $$La=\frac{4.4}{I_{D}/I_{G}}$$

### 2.7. Thermogravimetric−Differential Scanning Calorimetry (TG−DSC)

The thermogravimetric properties of pitches and fibers were measured using a STA 449 F5 thermal analyzer (Netzsch, Bavaria, Germany). The peak temperature (*T_p_*, °C), weight uptake (*ΔW*, %) and enthalpy change (*ΔH*, J) of PFs were collected from their TG−DSC curves in the air mood with a heating rate of 1 °C/min to 600 °C. The decomposition temperature (*T_d_*, °C), coking values (*CV*, %) at 1000 °C and *ΔH* of SFs were collected from their TG−DSC curves in the nitrogen mood with a heating rate of 5 °C/min to 1000 °C.

### 2.8. Mechanical, Electrical and Thermal Properties

The mechanical properties of CFs were determined by a XQ−1C single−filament machine (Shanghai New Fiber Instruments Co., Shanghai, China) with a gauge length of 20 mm according to the standard GB/T 31290−2014. The diameter (*D*) was visually observed under an YYS−80E optical microscope (MicroDemo, Beijing, China) to calculate the sectional area of fibers. The tensile modulus and tensile strength of CFs were calculated from the mean values of ten tests with the values distributing within 10%. The electrical resistance (*R*) of CFs was measured by four probe methods according to the GB/T 32993−2016 with an inner and outer gauge length of 25 mm (*L*)and 35 mm by a Model 580 micro−ohmmeter (Keithley, Cleveland, OH, USA). The electrical resistivity (*ρ*) and thermal conductivity (*λ*) of CFs was calculated by the following Equations (4) and (5) [16].

(4) $$\rho =\frac{\pi D^{2}R}{4L}$$

(5) $$\lambda =\frac{1261}{\rho }$$

## 3. Results and Discussion

### 3.1. Spinnability of Pitch Precursors and Properties of Pitch Fibers

**Figure 1:**
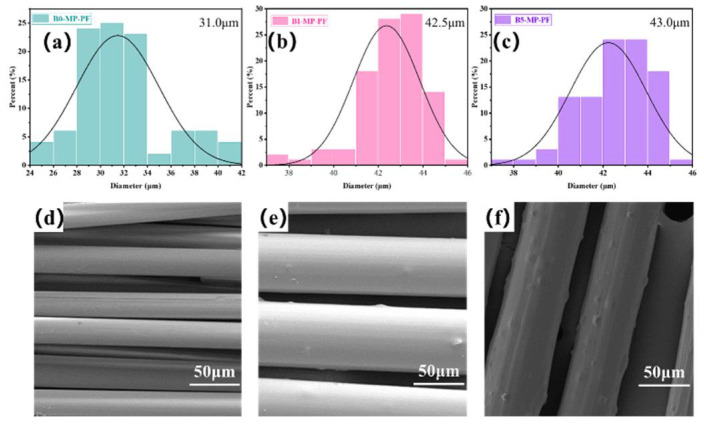
Fiber diameter distributions and SEM images of B0−MP−PF (**a**,**d**), B1−MP−PF (**b**,**e**) and B5−MP−PF (**c**,**f**).

Figure 1a–c shows the fiber diameter distributions of PFs prepared by the melt−spinning method. The average fiber diameters of B0−MP−PF, B1−MP−PF and B5−MP−PF, respectively, were 31.0, 42.5 and 43.0 μm, which indicated that the introduction of B_4_C would deteriorate the spinnability of pitch precursor, resulting in larger fiber diameter of PFs. Moreover, the SEM images of B0−MP−PF, B1−MP−PF and B5−MP−PF are showed in Figure 1d–f. It was noteworthy that there were many bulges on the surface along axial direction of B1−MP−PF and B5−MP−PF due to the dopped B_4_C nanoparticles while the surface of B0−MP−PF was smooth.

**Figure 2:**
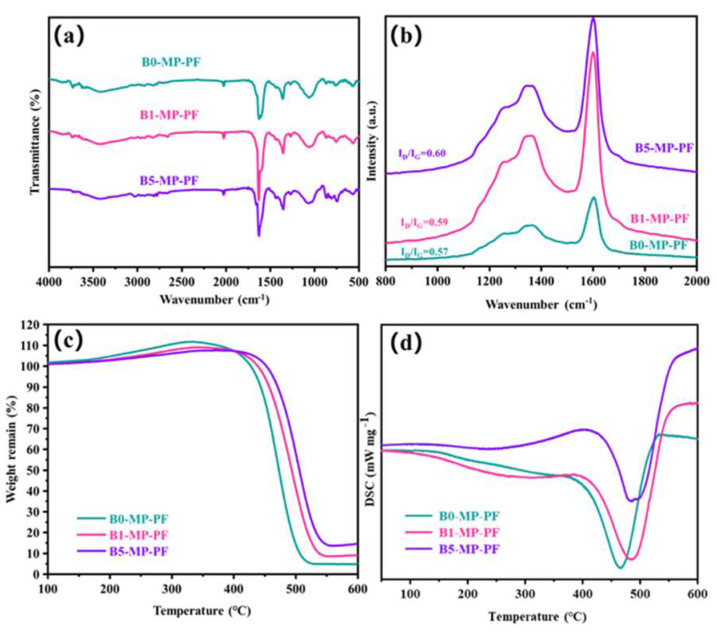
FTIR spectra (**a**), Raman spectra (**b**), and TG−DSC curves in air mood (**c**,**d**) of B0−MP−PF, B1−MP−PF and B5−MP−PF.

Figure 2a shows the FTIR spectra of PFs. As depicted in Figure 2a, B0−MP−PF, B1−MP−PF and B5−MP−PF had obvious characteristic peaks near 760, 1450 and 1620 cm^−1^. The vibration of C−H in the benzene ring around 760 cm^−1^ and the vibration of C = C in benzene ring near 1620 cm^−1^ indicated that there were a lot of aromatic hydrocarbons in the PFs. The vibration of methylene group (−CH_2_−C) in side chain near 1450 cm^−1^ indicated that the PFs also consisted of some aliphatic groups. Moreover, Table 1 shows the calculated *I_OS_* and *I_CHS_* values of PFs from FTIR spectra. Both of pristine and B_4_C−dopped PFs appeared almost the same *I_OS_* and *I_CHS_* values (*I_OS_* = 0.2475, *I_CHS_* = 0.5006 for B0−MP−PF; *I_OS_* = 0.2474, *I_CHS_* = 0.5005 for B1−MP−PF; *I_OS_* = 0.2473, *I_CHS_* = 0.5005 for B5−MP−PF), which proved that B_4_C had no effect on the aromaticity and aliphatic groups of pitch precursor. Furthermore, B1−MP−PF and B5−MP−PF had the similar peak positions in FTIR spectra, which demonstrated that B_4_C might be physically doped into the MP. Figure 2b shows the Raman spectra of B0−MP−PF, B1−MP−PF and B5−MP−PF. As depicted in Figure 2b, all PFs had two strong broadened peaks around 1300 and 1600 cm^−1^, respectively. Generally, the D peak at around 1335 cm^−1^ corresponds to defect lattice vibration mode, while the G peak at around 1584 cm^−1^ corresponds to an ideal graphite lattice vibration mode in the Raman spectra of carbon materials [17]. In addition, the A−band at 1500~1550 cm^−1^ was related to the functional groups [18,19]. Consequently, the area ratio of D and G bands (*I_D_/I_G_*) can be used to measure the orientation degree of pitch molecular, and the area ratio of A and G bands (*I_A_/I_G_*) can be used to quantify the functional groups of pitch molecules. In Table 1, the calculated *I_D_/I_G_* of PFs after doping B_4_C increased from 2.27 to 2.34, revealing that the addition of B_4_C would affect the orientation of pitch molecules, causing a poor spinnability of B_4_C−doped pitches. Additionally, the calculated *I_A_/I_G_* of PFs were the same of 0.15, indicating that the addition of B_4_C did not form new functional groups, which was consistent with the FTIR result.

### 3.2. Stabilization and Carbonization Behaviors of B_4_C−Doped Fibers and Pristine Fibers

Figure 2c,d shows the TG−DSC curve of PFs in air atmosphere with a heating rate of 1 °C/min to 600 °C to explore the stabilization behavior of PFs. Obviously, the B1−MP−PF and B5−MP−PF behaved a lower maximum weight gain (*ΔW*) of 9.23 and 8.19% than B0−MP−PF (12.12%) at 333 °C, which implied that the addition of B_4_C made the stabilization milder. Moreover, the peak temperature (*T_p_*) corresponding to the maximum oxidation weight gain in Table 1 were 333 °C for B0−MP−PF, 342 °C for B1−MP−PF and 380 °C for B5−MP−PF, respectively. The decrease of *ΔW* and the shift of *T_p_* to high temperature region of B_4_C−doped PFs during stabilization were mainly attributed the change of fibers microstructure under high boron concentration and the formation of blockage of boron oxide formed on the fibers surface to specific parts would inhibit the oxidation process [5]. As summarized in Table 1, the endothermic peaks are broadened and the enthalpy change (*ΔH*) has increased from 182 to 238 J. This contradiction between oxidation inhibition and more heat released by the reaction indicated that the addition of B_4_C had provided active sites for oxygen. Further oxygen preferentially entered interior fibers for internal reaction and made the reaction more uniform. A similar situation also occurred in the stabilization process of PAN−based fibers, the introduction of B_4_C inhibited the uneven distribution of oxygen in the skin and core of the fibers [11].

**Figure 3:**
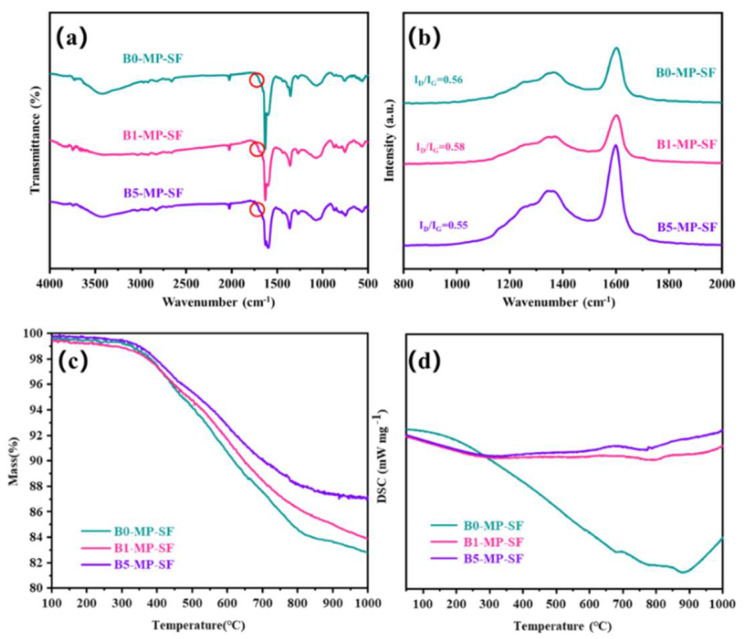
FTIR spectra (**a**), Raman spectra (**b**), and TG−DSC curves in nitrogen mood (**c**,**d**) of B0−MP−SF, B1−MP−SF and B5−MP−SF.

The FTIR spectra of SFs are showed in Figure 3a. The vibration absorption peaks of C−H near 760 and 1450 cm^−1^ decreased. As to the obtained SFs, the stabilization leaded to the appearance of new peaks at 1700 cm^−1^ corresponding to carbonyl (C=O) stretching vibration as shown in Figure 3a circles, indicating the existence of ketone, and carboxylate in the SFs. Meanwhile, compared to the PFs, *I_OS_* of SFs had significantly increased (*I_OS_* = 0.2489 for B0−MP−SF; *I_OS_* = 0.2484 for B1−MP−SF; *I_OS_* = 0.2486 for B5−MP−SF) and the *I_CHS_* had significantly reduced (*I_CHS_* = 0.5000 for B0−MP−SF; *I_CHS_* = 0.5002 for B1−MP−SF; *I_CHS_* = 0.5002 for B5−MP−SF) in Table 1, respectively, which meant that pitch branched chains were oxidized and benzene ring molecules undergo a series of reactions such as ring opening, forming oxygen bridge structure and connecting smaller planar molecular characteristics, resulting in the reduction of condensation degree and aromaticity. Furthermore, intercompared to the three SFs, it could be found that the rise of *I_OS_* and the decline of *I_CHS_* of B1−MP−SF and B5−MP−SF were lower than that of B0−MP−SF, less hydrogen was involved in the oxidation reaction revealing the mild of oxidative crosslinking reaction [20]. Figure 3b shows the Raman spectra of SFs. Notably, D and G peaks around 1300 and 1600 cm^−1^ of all SFs had decreased. Meanwhile, after the stabilization progress, the *I_D_/I_G_* of B0−MP−SF, B1−MP−SF, and B5−MP−SF were 2.16, 2.17 and 2.20, respectively (Table 1). These results revealed that the molecular arrangement in fibers were more regular due to oxidative cross−linking. And the *I_D_/I_G_* change rate of B1−MP−SF and B5−MP−SF were larger, illustrating that B_4_C played a beneficial role in oxidative cross−linking. In addition, the *I_A_/I_G_* of SFs also decreased (*I_A_/I_G_* = 0.10 for B0−MP−SF, *I_A_/I_G_* = 0.08 for B1−MP−SF, *I_A_/I_G_* = 0.07 for B5−MP−SF), which indicated that oxygen increased the content of functional groups of SFs. The larger change rate of *I_A_/I_G_* indicated that B_4_C in B1−MP−SF and B5−MP−SF had provided more active sites to increase the number of oxygen−containing functional groups in the corresponding SFs.

As shown in Figure 3c,d, the TG−DSC curve of SFs in nitrogen atmosphere with a heating rate of 5 °C/min to 1000 °C to explore the carbonization behavior of SFs. The TG curves simulated the weight loss of SFs in the carbonization stage. Significantly, the TG curves of B1−MP−SF and B5−MP−SF were above B0−MP−SF, and their coking value (*CV*) at 1000 °C were 82.34, 84.56 and 87.09%, respectively. This meant the addition of B_4_C leaded to an increase in carbonization yield and less gaseous products. Although the decomposition temperature (*T_d_*) of three SFs were similar about 360 °C, the *ΔH* of B0−MP−SF (794 J) was evidently higher than that of B1−MP−SF (108 J) and B5−MP−SF (104 J) in Table 1. Compared with B1−MP−SF and B5−MP−SF, most of oxygen in B0−MP−SF existed on the surface of fibers and could be more easily removed in the carbonization stage and gave off a lot of heat, which also indirectly confirmed that the B_4_C nanoparticles mentioned above made the fibers oxidation more uniform.

After carbonization, *I_OS_* and *I_CHS_* of CFs were conspicuously reduced (*I_OS_* = 0.2472, *I_CHS_* = 0.4992 for B0−MP−CF−1000, *I_OS_* = 0.2469, *I_CHS_* = 0.4996 for B1−MP−CF−1000, *I_OS_* = 0.2470, *I_CHS_* = 0.4998 for B5−MP−CF−1000) in Table 1, which suggested that high temperature carbonization could remove large amount of non−carbon elements and reduce the number of methyl (−CH_3_) and methylene (−CH_2_−) on the benzene rings In this situation, the degree of condensation and aromaticity had increased due to pitch molecules continued to shrink into larger planar molecules. In addition, the changes of *I_OS_* and *I_CHS_* of B1−MP−SF and B5−MP−SF were lower than that of B0−MP−SF. Implying that the removal of non−carbon elements was more difficult and the branch chain was more difficult to break. This was because that the stabilization of B1−MP−CF−1000 and B5−MP−CF−1000 were more evenly, and the synergistic decarboxylation of ester and anhydride crosslinking would further improve the stability of pitch molecules, which led to cage aromatic radicals, enabling them to better position in the recombination without migration or rearrangement [21]. Moreover, the *I_D_/I_G_* of CFs in Table 1 (*I_D_/I_G_* = 2.58 for B0−MP−SF, *I_D_/I_G_* = 3.14 for B1−MP−SF, *I_D_/I_G_* = 3.36 for B5−MP−SF) increased, indicating that a large amount of gas was produced at carbonization temperature, causing certain crystallite defects during carbonization process. Meanwhile, it is worth noting that there were many amorphous carbon structures in CFs. The larger *I_D_/I_G_* change rate exhibited that the B_4_C in B1−MP−CF and B5−MP−CF would cause more defects in fibers during carbonization process. The *I_A_/I_G_* of B0−MP−CF−1000, B1−MP−CF−1000 and B5−MP−CF−1000 were 0.12, 0.09 and 0.08, respectively. The lower *I_A_/I_G_* change rate revealed that the B_4_C in B1−MP−CF and B5−MP−CF made the existence of functional groups more stable and less release of gaseous products in the carbonization process.

### 3.3. Structures and Properties of B_4_C−Doped Carbon Fibers and Pristine Carbon Fibers

**Figure 4:**
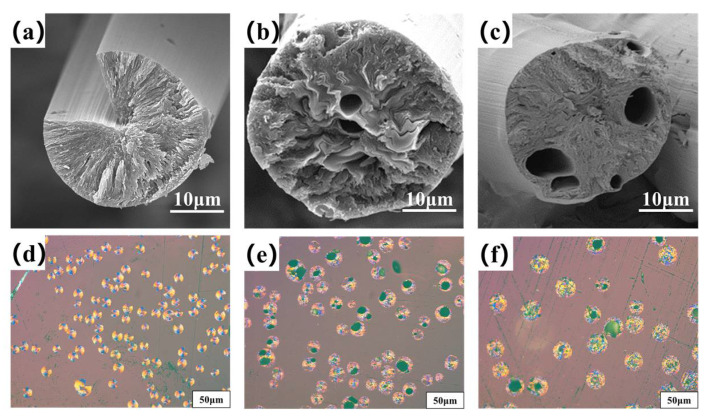
SEM images and POM photos of B0−MP−CF−1000 (**a**,**d**), B1−MP−CF−1000 (**b**,**e**) and B5−MP−CF−1000 (**c**,**f**).

Figure 4a–c are the transversal surface SEM images of B0−MP−CF−1000, B1−MP−CF−1000 and B5−MP−CF−1000. As depicted, B0−MP−CF−1000 presented a splitting structure with a wedge−shaped splitting angle of 60°. This was attributed to the radial shrinkage of fibers and the stress was concentrated in the center of the circle. It could be found that there was still a disordered carbon structure inside B0−MP−CF−1000 before the high−temperature graphitization treatment. At the same time, the SEM images intuitively reflected that the splitting microstructure disappeared while many holes and appeared in B1−MP−CF−1000 and B5−MP−CF−1000. Moreover, the graphite microcrystallite of B1−MP−CF−1000 showed a highly folded structure, while the lamellar structure of B5−MP−CF−1000 graphite microcrystallite was inconspicuous. Meanwhile, it was obvious that the B1−MP−CF−1000 and B5−MP−CF−1000 appeared to have skin−core structural problems, which indicate that the B_4_C−dopped SFs were not fully stabilized. These discrepancies were attributed to the structure change of CFs ascribe to the introduction of B_4_C. Figure 4d–f are the POM photos of B0−MP−CF−1000, B1−MP−CF−1000 and B5−MP−CF−1000. Apparently, the different colors of the CFs transversal surface represented a spatial orientation of graphite microcrystals, which exhibited an optical anisotropy. Furthermore, it was confirmed that the introduction of B_4_C leaded to the formation of holes and other defects in B_4_C−doped CFs.

**Figure 5:**
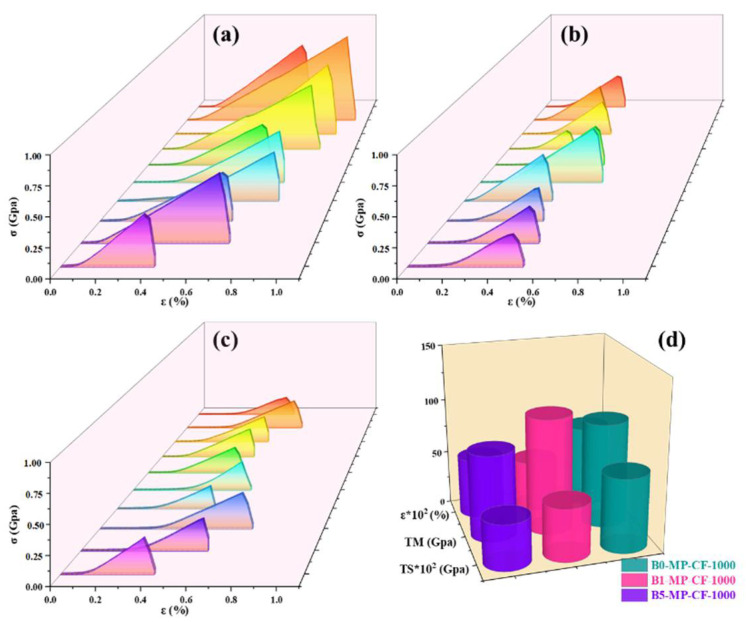
Stress−strain curves of B0−MP−CF−1000 (**a**), B1−MP−CF−1000 (**b**), B5−MP−CF−1000 (**c**), and their corresponding mechanical properties (**d**).

Figure 5 shows the stress−strain curves of B0−MP−CF−1000, B1−MP−CF−1000, B5−MP−CF−1000 and their corresponding mechanical properties of CFs. It was prominent that B0−MP−CF−1000 with the fiber diameter of 30 μm showed the optimal mechanical properties with tensile strength of 666 MPa, tensile modulus of 95 GPa and elongation of 0.73%. Oppositely, B1−MP−CF−1000 and B5−MP−CF−1000 showed the poor tensile strength of 475 and 412 MPa, and low tensile modulus of 105 and 78 GPa, which might be related to cracked structure, micropore and hollow structure. These results suggested that B_4_C could improve the splitting microstructure of CFs, but the introduction of defects would decrease the mechanical properties of ones.

**Figure 6:**
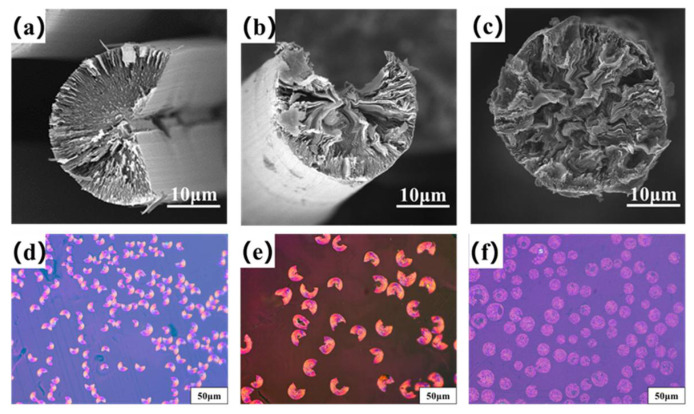
SEM images and POM photos of B0−MP−CF−3000 (**a**,**d**), B1−MP−CF−3000 (**b**,**e**) and B5−MP−CF−3000 (**c**,**f**).

Figure 6 shows the transversal surface SEM images and POM photos of B0−MP−CF−3000, B1−MP−CF−3000 and B5−MP−CF−3000. It was worthwhile to mention that CFs obtained from the graphitization of 3000 °C exhibited more complex cross−sectional properties. For the cross−section, the B0−MP−CF−3000 showed a good graphite microcrystalline orientation. Additionally, the graphite sheets of B1−MP−CF−3000 and B5−MP−CF−3000 were apparent larger. Similar conclusions could be drawn from POM photos, and B1−MP−CF−3000 orientation was better than B5−MP−CF−3000. Moreover, compared with B5−MP−CF−3000, B1−MP−CF−3000 had fewer defects and better arrangement of graphite sheets. The results showed that B_4_C could indeed catalyze graphitization and obtain larger graphite crystallites in CFs.

The *λ* of CFs at different graphitization temperatures of 2300, 2600, 2800 and 3000 °C are summarized in Table 2. Apparently, the *λ* of B1−MP−CF_S_ and B5−MP−CF_S_ were much higher than that of B0−MP−CF_S_. This demonstrated that B_4_C played a crucial role in catalytic graphitization at 2300 °C, which highly improved *λ* of CFs. Subsequently, it could be noticed that the thermal conductivity of B1−MP−CF_S_ and B5−MP−CF_S_ increased fastest in the temperature range of 2600~2800 °C, revealing that B_4_C decomposed at this temperature range. The free boron atoms entered the hexagonal graphite grid, improving the microstructure and the orientation of graphite microcrystallite. In this case, the growth rate of thermal conductivity of CFs were conspicuously improved. Nevertheless, the excessive doping could lead to more defects in B5−MP−CF_S_, thereby the λ of them were slightly low. More importantly, the catalytic graphitization of B_4_C markedly reduced the graphitization temperature. The *λ* of B0−MP−CF−3000 prepared at graphitization of 3000 °C was only 659 W/m•K. Surprisingly, the *λ* of B1−MP−CF−2300 prepared at graphitization temperature of 2300 °C was 681 W/m•K, and that of B1−MP−CF−3000 prepared at graphitization temperature of 3000 °C was significantly larger with 1051 W/m•K. Moreover, the thermal conductivity of boron doped CFs at 2600 °C exceeded that of most high properties CFs at 3000 °C prevailing in the market, which implied the graphitization temperature had reduced preliminarily. Meanwhile, several commercial MPCFs with high thermal conductivity (XN−90, K13C2U, K13D2U, K1100) were selected to compare their microcrystalline parameters and electrothermal properties [2]. It was noted that the λ of B1−MP−CF−2600 was obviously higher than that of XN−90, K13C2U or K13D2U, and λ of B1−MP−CF−3000 was very near to the K1100, which furtherly indicated the MPCFs with high thermal conductivity could be achieved by the in-situ doping B_4_C nanoparticles in MP.

**Figure 7:**
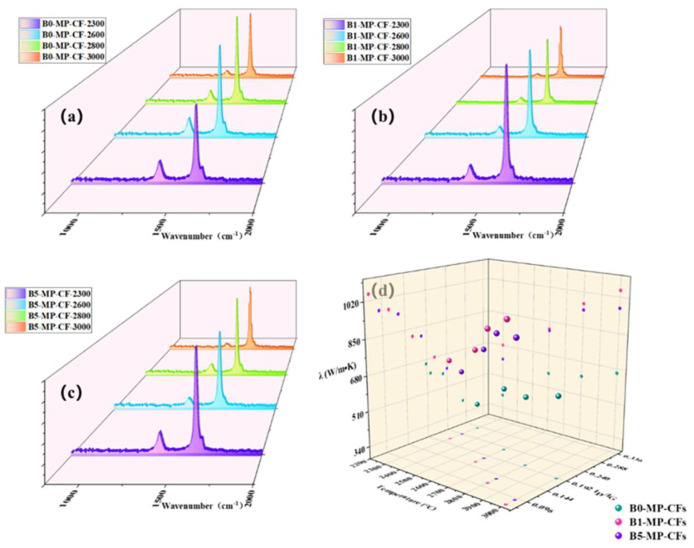
Raman spectra of CFs at different graphitization temperatures of 2300, 2700, 2850, 3000 °C (**a**–**c**), and scatter plot of CFs at different temperatures, I_D_/I_G_ and λ (**d**).

Actually, the influence of thermal conductivity on CFs can be connected with the microstructure, graphite microcrystalline transformation and orientation of CFs. Figure 7a–c shows Raman spectra of CFs at different graphitization temperatures of 2300, 2600, 2800, 3000 °C. It could be observed that the intensity of the D peak at 1335 cm^−1^ was significantly weakened, while the G peak at 1584 cm^−1^ was sharpened, and the A−band at 1500~1550 cm^−1^ was weakened or even disappeared. Additionally, the band at around 1620 cm^−1^ named as D’−band was related to disordered graphite structure after graphitization [18,19]. The *I_D_/I_G_* (average of four values) and *I_D’_/I_G_* values of B1−MP−CF_S_ and B5−MP−CF_S_ were lower than that of B0−MP−CF_S_ in Table 2, indicating that B_4_C had a significant effect on catalytic graphitization, which improved the graphitization degree of the CFs and promoted the transformation of CFs from amorphous carbon to regular graphite microcrystallite. As depicted in Figure 7d, the λ value of B1−MP−CFs were the highest at different temperatures. In addition, B_4_C had a momentous effect on thermal conductivity than that of graphitization temperature. Therefore, it could be basically determined that the catalytic graphitization of B_4_C played a vital role in improving the thermal conductivity of fibers and reducing the graphitization temperature. Interestingly, there seemed to be a negative correlation between *λ* and *I_D_/I_G_*. To further understand the relationship between the two paraments, a regression analysis was performed and presented in Table 2. A linear relationship (*R^2^* = 0.94) between them was obtained by the linear fitting method through the Excel software based on the average data of each sample in Table 2, which demonstrated a conspicuous negative correlation between *I_D_/I_G_* and *λ*. This linear regression analysis provided a new idea for calculating the *λ* of MPCFs by *I_D_/I_G_* from Raman spectra.

Apart from that, it was necessary to find the mechanism corresponding to the *λ* of CFs at the microscopic level. Generally, heat conduction was mainly achieved by phonons, and the transfer effect of phonons would directly affect the magnitude of *λ*. As to MPCFs, the main factor affecting the transmission of phonons was the change of grain size. The calculation results are collected in Table 2. Corresponding to as the lattice size (*L_a_*), the λ showed an upward trend. This was because the graphite microcrystallite had grown up, and the amount of crystal boundaries and the probability of phonon scattering in CFs decreased. In addition, B1−MP−CF_S_ had the largest crystallite size at each graphitization temperature, which further confirmed that B_4_C played an excellent role in catalytic graphitization, promoted the growth of graphite microcrystallite, and made CFs obtained the higher *λ*.

### 3.4. Influence Mechanism of B_4_C Nanoparticles on the Structural Evolution of Carbon Fibers

**Figure 8:**
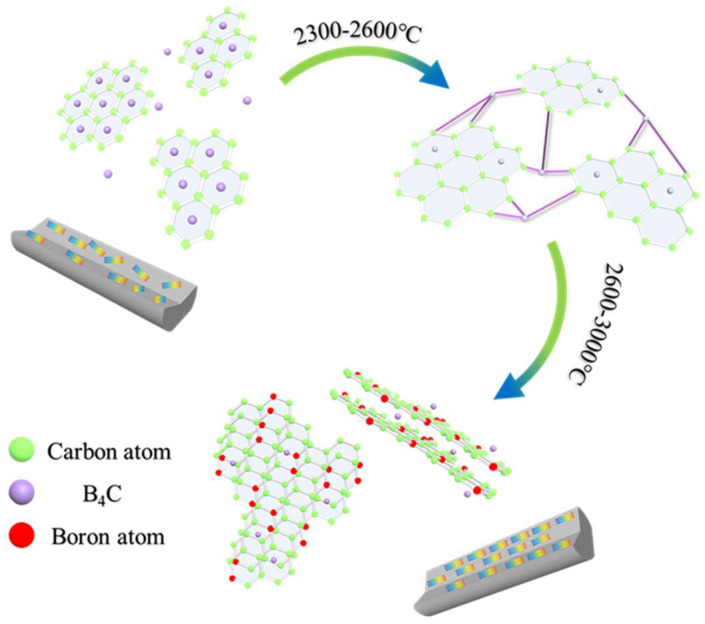
Catalysis and transformation of B_4_C in the graphitization stage.

Based on the previous results and analysis, the mechanism and evolution of B_4_C in MPCFs was reasonably deduced as showed in Figure 8. According to the characterization analysis of pitch precursors and PFs, the B_4_C nanoparticles were uniformly dispersed in PFs by physical doping after in-situ doping and melt−spinning processes. Subsequently, most of the boron element still existed in the form of B_4_C during stabilization and carbonization processes because B_4_C had excellent thermal stability and extremely high melting point [4,6,11]. In addition, as plotted in the TG−DSC curve of PFs, B_4_C provided active sites for oxygen, allowing it to enter the interior of PFs, which promoted the stabilization more uniform. When the carbonization temperature was 1000 °C, B_4_C existed in the graphite chaotic layer structure or in the lattice gap. Meanwhile, a large amount of non−carbon elements such as oxygen were removed at this stage, and defects such as lattice distortion began to appear. Therefore, the mechanical properties of B1−MP−CF−1000 and B5−MP−CF−1000 decreased compared with B0−MP−CF−1000. In addition, scholars also confirmed that the dissolution and precipitation of carbon could be completed by the conversion of carbides [3]. B_4_C dissolved disordered carbon to form carbides, and boron atoms would leave the interstitial positions of the graphite network structure when the graphitization temperature increased over 2300 °C. Then boron atoms would spontaneously move to the grid points of the hexagonal grid of graphite crystals [10], and played a role of catalytic graphitization. During the graphitization stage, B_4_C in the gap or between graphite layers would play a role of a bridge. Its specific function was to connect the surrounding randomly disperse graphite flakes, achieving the dissolution of disordered carbon. As the graphitization temperature was further increased, B_4_C decomposed and free boron atoms appeared, ascribe to gaining kinetic energy under the action of heat energy to move to the substitution position. The structure was relieved, and the degree of graphitization was improved. This conjecture appropriately explained the reason why in-situ doping B_4_C in pitch could prepare higher thermal conductivity CFs at lower graphitization temperature.

## 4. Conclusions

MPCFs with high thermal conductivity were achieved by the in-situ doping B_4_C nanoparticles in MP. During the heat treatment stage of fibers, B_4_C nanoparticles acted as active sites, which were beneficial to the preferentially entrance of oxygen into the PFs in the stabilization process, realizing more uniform stabilization. In the carbonization stage, B_4_C nanoparticles had improved the carbonization yield of CF_S_ and decreased gaseous products, but the mechanical properties of CFs were reduced due to the formation of defects from the B_4_C introduction. In addition, a linear relationship (R^2^ = 0.95) between *I_D_/I_G_* and *λ* was found, providing a new idea for calculating the thermal conductivity of MPCFs. Most significantly, B_4_C could effectively promote the graphitization degree of fibers and the growth of graphite microcrystallite, which thereby played a crucial role in catalyzing graphitization and declining graphitization temperature. Accordingly, it would provide theoretical guidance for reducing energy consumption while prolonging the service lives of equipment, which could be conducive to instruct the preparation of high thermal conductivity CFs in the industrial production.

## Figures and Tables

**Table 1:** General properties of PFs, SFs and CFs.

Samples	TG−DSC			FTIR		Raman		Yield (%)
	*T_p_(T_d_)* (°C)	*ΔW (CV)* (%)	*ΔH* (J)	*I_OS_*	*I_CHS_*	*I_D_/I_G_*	*I_A_/I_G_*	
B0−MP−PF	333	12.12	182	0.2475	0.5006	2.27	0.15	−
B1−MP−PF	342	9.23	238	0.2474	0.5005	2.29	0.15	−
B5−MP−PF	380	8.19	232	0.2473	0.5005	2.34	0.15	−
B0−MP−SF	359	82.34	794	0.2489	0.5000	2.16	0.10	114
B1−MP−SF	361	84.56	108	0.2484	0.5002	2.17	0.08	110
B5−MP−SF	363	87.09	104	0.2486	0.5002	2.20	0.07	108
B0−MP−CF−1000	−	−	−	0.2472	0.4992	2.58	0.12	81
B1−MP−CF−1000	−	−	−	0.2469	0.4996	3.14	0.09	84
B5−MP−CF−1000	−	−	−	0.2470	0.4998	3.36	0.08	86

**Table 2:** Microcrystalline parameters and electrothermal properties of CFs.

Samples	*I_D_/I_G_*	*I_D’_/I_G_*	*L_a_* (nm)	*ρ* (μΩ·m)	*λ* (W/m•K)
B0−MP−CF−2300	0.2800	0.21	15.71	3.09	408
B1−MP−CF−2300	0.2100	0.16	20.95	1.85	681
B5−MP−CF−2300	0.2425	0.14	18.33	2.08	606
B0−MP−CF−2600	0.2225	0.19	19.13	2.16	584
B1−MP−CF−2600	0.1550	0.15	27.50	1.55	812
B5−MP−CF−2600	0.1700	0.12	24.44	1.57	803
B0−MP−CF−2800	0.2000	0.17	22.00	2.08	606
B1−MP−CF−2800	0.1075	0.10	40.00	1.31	964
B5−MP−CF−2800	0.1400	0.13	33.85	1.35	934
B0−MP−CF−3000	0.1875	0.12	23.16	1.91	659
B1−MP−CF−3000	0.0700	0.11	62.86	1.20	1051
B5−MP−CF−3000	0.0800	0.11	48.89	1.30	968
XN−90	0.14	−	38.70	2.95	428
K13C2U	0.12	−	46.00	2.07	609
K13D2U	0.07	−	50.61	1.58	799
K1100	0.09	−	70.30	1.15	1099
Fitting equation	λ = 1261.9 − 2938.2 ∙ I_D_/I_G_ (R^2^ = 0.94)				

## Data Availability

Data is contained within the article.

## Sample Availability

Samples of the compounds are not available from the authors.
